# Psychiatric Implications of Genetic Variations in Oligodendrocytes: Insights from hiPSC Models

**DOI:** 10.3390/life15060921

**Published:** 2025-06-06

**Authors:** Martina D’Angelo, Valeria Di Stefano, Ilaria Pullano, Francesco Monaco, Luca Steardo

**Affiliations:** 1 Department of Health Sciences, University of Catanzaro Magna Graecia, 88100 Catanzaro, Italy; martina.dangelo001@studenti.unicz.it (M.D.); valeria.distefano@studenti.unicz.it (V.D.S.); pullano.ilaria@gmail.com (I.P.); 2 Department of Mental Health, Azienda Sanitaria Locale Salerno, 84132 Salerno, Italy; fmonaco1980@gmail.com; 3 European Biomedical Research Institute of Salerno (EBRIS), 84125 Salerno, Italy

**Keywords:** oligodendrocyte precursor cells, psychiatric disorders, myelination, neuron–glia interaction, neurodevelopment

## Abstract

Oligodendrocyte precursor cells (OPCs) are a dynamic and heterogeneous population of glial cells essential for brain development and myelination. Beyond their well-established role in oligodendrogenesis, emerging evidence suggests that OPCs contribute to synaptic regulation, neuronal communication, and brain plasticity. Recent studies have increasingly implicated OPC dysfunction in the pathophysiology of psychiatric disorders, particularly schizophrenia (SCZ), bipolar disorder (BD), and major depressive disorder (MDD). This narrative review integrates clinical, genetic, transcriptomic, and histological findings to examine the role of OPC alterations in mental illnesses. In SCZ, OPC abnormalities predominantly affect myelination, but recent data also suggest deficits in non-canonical functions, including neuron–OPC communication. Findings in BD largely mirror those in SCZ, implying shared OPC-related mechanisms across these disorders. In contrast, OPC involvement in MDD appears more complex, with evidence supporting both myelination deficits and non-canonical dysfunctions, such as impaired neuro–glial interactions and perineuronal network alterations. The developmental timing of OPC dysfunction may represent a common denominator underlying psychiatric disorders, as early-life stress and neurodevelopmental disturbances have been linked to OPC impairments. Moreover, given the shared developmental origins of OPCs and parvalbumin-positive interneurons, disruptions in their mutual interactions may contribute to broader neural network dysregulation. Despite these insights, the field remains in its infancy. Future studies integrating independent human cohorts with robust preclinical models are needed to clarify the extent of OPC involvement in psychiatric pathophysiology. Understanding OPC dysfunction may reveal novel biomarkers and open new avenues for individualized therapeutic interventions and preventive strategies in mental health.

## 1. Introduction

Mental disorders affect millions of individuals worldwide, irrespective of cultural background, gender, or ethnicity [1]. With an estimated global lifetime prevalence of 25–30%, these conditions represent a significant burden [2], leading to impaired daily functioning, disrupted social relationships, and reduced quality of life for both affected individuals and their families. Despite their widespread occurrence, effective pharmacological treatments remain limited, and many patients exhibit inadequate therapeutic responses [3]. Addressing this challenge requires a thorough understanding of the cellular and molecular mechanisms underlying the onset and progression of psychiatric disorders. Over the past few decades, psychiatric research has shifted from a predominantly neuron-centered perspective to a more integrative approach, recognizing mental disorders as dysregulations of complex brain networks involving multiple cellular and molecular components.

Recent findings suggest that oligodendrocyte precursor cells (OPCs) may be among the key biological substrates contributing to the pathophysiology of psychiatric conditions [4,5]. OPCs, also known as oligodendrocyte progenitors or NG2-glia, are a population of non-neuronal cells distributed throughout the central nervous system (CNS) [5]. They express distinct molecular markers, such as platelet-derived growth factor receptor alpha (PDGFRα) and neuron-glial antigen 2 (NG2) proteoglycan, which differentiate them from mature oligodendrocytes and other glial populations [6]. In mammalian development, OPCs originate from both ventral and dorsal regions of the embryonic spinal cord and forebrain, while in humans they also arise from the outer subventricular zone [7]. A subset of OPCs differentiates into mature oligodendrocytes during early development, whereas others retain their progenitor properties, allowing them to proliferate, migrate, and persist in the CNS throughout adulthood [6,8]. Traditionally, OPCs have been considered a reservoir of progenitor cells that replenish oligodendrocytes and maintain myelination. However, significant heterogeneity has been observed in their proliferation, differentiation, and cell cycle dynamics, particularly when comparing OPCs in gray matter versus white matter [9].

Although OPCs’ well-established role in myelination is critical for neural function and maintenance, emerging preclinical studies suggest that these cells perform additional, non-canonical functions within the brain [10]. These include regulating synaptic activity, responding to neural injury, modulating immune responses, maintaining blood–brain barrier integrity, and stabilizing extracellular potassium levels [8,11]. OPCs are highly sensitive to changes in their microenvironment and can undergo proliferation, migration, differentiation, and myelination in response to various stimuli, including neuronal activity [11,12,13,14,15,16]. Moreover, they receive direct synaptic inputs from both GABAergic and glutamatergic neurons [13,17,18], and recent evidence indicates that OPCs may form synaptic connections with GABAergic neurons, influencing their function [19]. Additionally, oligodendrogenesis and OPC activity have been implicated in motor, sensory, and cognitive processes, further underscoring their broad functional diversity in brain physiology [20,21,22,23,24,25].

Given their dynamic nature, responsiveness to neural signals, and involvement in key behavioral processes, OPCs have increasingly attracted attention in psychiatric research. Growing evidence suggests that OPC dysfunction may play a role in the pathogenesis of mental illnesses [5,26]. However, despite the expanding interest in this area, a comprehensive synthesis of findings related to OPC involvement in psychiatric disorders, particularly in human studies, remains lacking. Therefore, this narrative review aims to provide an updated and integrative perspective on the role of OPCs in psychiatric conditions. Identifying shared pathological mechanisms involving both canonical and non-canonical OPC functions and highlighting existing knowledge gaps could offer crucial insights with which to guide future research. This may, in turn, foster the development of novel therapeutic approaches for mental disorders and support preventive strategies aimed at preserving mental health.

## 2. OPC Alterations in Severe Mental Disorders

For decades, disruptions in the oligodendrocyte lineage and abnormalities in myelin structure have been documented in individuals diagnosed with psychiatric, neurodevelopmental, and neurodegenerative disorders [27]. However, it was not until the early 21st century, with the advent of high-throughput and unbiased screening technologies, that psychiatry moved beyond the long-standing assumption that equated brain dysfunction solely with neuronal impairment. This paradigm shift led to a growing interest in the role of oligodendrocyte lineage cells in the onset and progression of mental illnesses.

A landmark study by Hakak et al. [28] provided compelling evidence of the reduced expression of oligodendrocyte-related genes in the dorsolateral prefrontal cortex of individuals with schizophrenia (SCZ) compared to healthy controls. This unbiased transcriptomic analysis is now considered a pivotal moment in SCZ research, as it helped redefine the conceptual framework of psychiatric disorders.

Following this discovery, numerous research groups began investigating additional oligodendrocyte-related markers in independent cohorts of individuals with SCZ, as well as in those diagnosed with other psychiatric conditions, such as major depressive disorder (MDD) and bipolar disorder (BD) [29,30].

Of relevance to this narrative review, the earliest evidence specifically linking OPCs to psychiatric disorders dates back to 2003. In a study by Tkachev et al. [31], gene expression changes were analyzed in Brodmann area (BA) 9 of post mortem brain samples from individuals with SCZ and BD by using microarray technology. Although OPCs were not the primary focus, this study represents one of the first to quantify OPC-specific markers in human post mortem samples from psychiatric patients. The following sections will systematically present OPC-related findings within the framework of conventional psychiatric diagnostic categories.

## 3. Schizophrenia

SCZ is a complex, polygenic psychiatric disorder with an estimated lifetime prevalence of approximately 1% worldwide. Epidemiological studies indicate a slightly higher incidence in males than in females. The disorder typically manifests in late adolescence or early adulthood, and is characterized by a range of symptoms, including hallucinations, delusions, emotional blunting, and cognitive impairments [32,33]. Despite extensive research, the precise etiology of SCZ remains elusive, with considerable heterogeneity in symptom presentation and severity across individuals. Multiple cellular and molecular mechanisms have been proposed to contribute to the disease pathogenesis, including alterations in mature oligodendrocytes and myelin integrity [34].

Although less extensively studied, OPCs have also been implicated in SCZ pathology. In the following sections, we summarize key findings related to OPC involvement in SCZ, focusing on (1) genetic variations and mutations, some of which have been validated through human-induced pluripotent stem cell (hiPSC) models, (2) alterations in gene expression, and (3) histopathological evidence of OPC abnormalities in post mortem brain samples.

## 4. Insights into Genetic Variations Through hiPSC Models

Research on both rare and common genetic variants has provided evidence that OPC dysfunction may contribute to SCZ pathophysiology. A genome-wide linkage disequilibrium analysis conducted on a cohort of 175 families, each with at least two members diagnosed with SCZ or another psychotic disorder, identified a significant linkage region at chromosome 15q22-24. This region includes the gene encoding chondroitin sulfate proteoglycan 4 (CSPG4), also known as NG2 [35,36]. Additionally, several rare missense mutations within CSPG4/NG2 have been associated with SCZ, with the CSPG4A131T variant found to segregate with the disorder. Notably, individuals carrying this rare mutation were either diagnosed with SCZ or had a history of psychiatric illnesses [37].

A study employing hiPSC technology examined the functional consequences of this mutation in patient-derived neuronal and glial cell lines. Interestingly, while iPSC-derived neurons from CSPG4A131T carriers exhibited only minor differences in input resistance and the action potential threshold compared to controls, OPCs derived from the same individuals displayed several abnormalities [37]. These included a reduced cell size, defective post-translational processing of the NG2 protein, and protein folding anomalies. Furthermore, aberrant subcellular localization of NG2 was observed, as evidenced by increased co-localization with calreticulin, an endoplasmic reticulum marker. These cellular changes were accompanied by a general decline in oligodendrogenesis and OPC viability [37]. In parallel, magnetic resonance imaging (MRI) analyses of SCZ patients carrying the CSPG4A131T mutation revealed decreased global fractional anisotropy (FA) and increased microstructural alterations in white matter, both indicative of myelin abnormalities. These findings suggest that oligodendrocyte lineage dysfunction, rather than neuronal deficits, may be central to the etiology of this particular form of familial SCZ [37].

Further evidence for the role of OPCs in SCZ has emerged from independent iPSC-based studies. One investigation reported a significant reduction in the number of O4+ oligodendrocyte lineage cells derived from SCZ patients’ iPSCs compared to controls [38]. Notably, none of the patients in this study carried rare CSPG4 variants, suggesting that OPC involvement in SCZ extends beyond genetically rare forms of the disorder [39]. Moreover, in SCZ patients, the percentage of O4+ cells positively correlated with the magnetization transfer ratio (MTR), a conventional MRI measure of myelin content, in frontal white matter. This correlation was absent in healthy controls, implying that impaired OPC differentiation in vitro may underlie the observed deficits in white matter integrity in SCZ patients [38].

Another study using a familial SCZ model provided additional support for the involvement of the oligodendrocyte lineage in disease pathology. Windrem et al. [40] generated human glial progenitor cells (hGPCs) from SCZ patient-derived iPSCs and transplanted them into the brains of Shiverer mice, which lack myelin basic protein (MBP) and exhibit severe CNS hypomyelination [39]. Compared to control-derived hGPCs, some SCZ-derived hGPCs displayed impaired migratory capacity, failing to colonize the white and gray matter efficiently. This was accompanied by hypomyelination and the reduced expression of synaptic markers. The authors hypothesized that these deficits may be driven by impaired neuron–glia synaptic interactions [40]. However, not all SCZ-derived hGPCs exhibited these abnormalities, further emphasizing the heterogeneity of the disorder [40].

Beyond rare genetic variants, studies investigating polygenic risk scores (PRSs) have also implicated OPCs in SCZ. Papiol et al. [41] demonstrated that an OPC-specific PRS, but not a PRS for mature oligodendrocytes, was linked to structural brain changes in the left CA4/Dentate Gyrus of the hippocampus in SCZ patients undergoing aerobic exercise. Specifically, individuals with a higher genetic risk burden related to OPCs exhibited a reduced increase in CA4/Dentate Gyrus volume following high-intensity exercise. These findings suggest that OPC-related genetic susceptibility may influence hippocampal plasticity in a subset of SCZ patients, potentially affecting their responsiveness to non-pharmacological interventions [41].

In another study integrating genetic, transcriptomic, and neuroimaging data, researchers proposed that regional cortical thinning in SCZ is modulated by cell-type-specific genetic predispositions [42]. Interestingly, higher OPC-related genetic risk scores were associated with less-pronounced cortical thinning in SCZ, highlighting the complexity of genetic influences on the brain structure. While further validation is needed, these findings reinforce the notion that OPC dysfunction contributes to the heterogeneity of SCZ and may play a key role in its underlying neurobiology.

## 5. Gene Expression and Protein Alterations

The genetic variability observed in SCZ is also reflected in post mortem studies analyzing gene expression patterns. Findings regarding the expression of CSPG4/NG2 transcripts have been inconsistent across different brain regions. For instance, one study reported an upregulation of CSPG4/NG2 mRNA levels in the putamen [29], while another found no significant changes in Brodmann area 9 (BA9) [31] when comparing SCZ patients with matched controls. Additionally, two independent investigations revealed a downregulation of OLIG1, OLIG2, and SOX10 transcripts across multiple cortical areas, including BA9, in SCZ cases relative to controls [31,43].

These transcriptional alterations are likely associated with mature oligodendrocytes rather than OPCs, as the expression levels of OPC-specific markers NG2 and PDGFRA remained unchanged. In contrast, several myelin-related genes were significantly downregulated in SCZ samples [31,43]. However, it is important to acknowledge that other studies failed to fully replicate these findings [44,45]. Some researchers have suggested that variations in oligodendrocyte lineage-related gene expression may be influenced by individual genetic predispositions [31], while others have proposed that the observed alterations could be mediated, at least in part, by the effects of antipsychotic medications [45].

Further evidence of OPC involvement in SCZ comes from studies examining transcripts and proteins critical for OPC function, although these molecules are not exclusively expressed in OPCs. One investigation employing bottom-up shotgun mass spectrometry identified disruptions in the Ephrin-B signaling pathway within the corpus callosum of SCZ patients [46]. Specifically, alterations were found in guanine nucleotide-binding protein (GNB4) and VAV guanine nucleotide exchange factor 2 (VAV2), both of which are involved in neuron–OPC interactions and may play a role in OPC migration [46]. However, as these markers are also highly expressed in microglia, astrocytes, and neurons, it remains unclear whether these pathway alterations predominantly affect OPCs or have a broader impact on other glial and neuronal populations [47].

Additionally, a computational analysis integrating multiple bulk RNA-seq datasets revealed a reduction in specific isoforms of Discoidin Domain Receptor Tyrosine Kinase 1 (DDR1) transcripts in the dorsolateral prefrontal cortex of SCZ patients. Given that *DDR1* is enriched in OPCs and regulates cell morphology changes during the cell cycle, these findings suggest a potential role for DDR1 in the impaired maturation and proliferation dynamics of OPCs in SCZ [48]. Similarly, another study reported a correlation between OPC marker expression and cell cycle-related genes in the internal capsule of SCZ patients, suggesting a possible slowdown in oligodendrocyte lineage turnover compared to controls [49].

Although these results point to a disruption in OPC cell cycle dynamics and differentiation in SCZ, some methodological considerations should be noted. For instance, the post mortem interval (PMI) and age of SCZ samples in the study by Kerns et al. [49] differed significantly from those of control subjects, potentially influencing the observed gene expression changes. As a result, while these findings provide valuable insights into the role of OPCs in SCZ, they should be interpreted with caution, and further studies are needed to validate and refine these observations.

## 6. Histological Alterations in Schizofrenia

Histological analyses of post mortem brain tissue have provided further insights into potential OPC abnormalities in SCZ. Kolomeets et al. conducted Nissl staining on brain samples from SCZ patients and matched controls, quantifying OPC density (identified as oligodendrocyte clusters) and mature oligodendrocytes in both the parietal cortex (Brodmann areas 39 and 40) and the putamen [50,51]. Their findings revealed that individuals with adolescent-onset SCZ exhibited a reduction in OPC density in layer 3 of BA39 and BA40, while in adult-onset cases the reduction was confined to layer 3 of BA39 [50]. Additionally, they identified a sex-specific difference in the putamen, where male SCZ patients, but not females, displayed a significant decrease in OPC density [51].

Conversely, studies examining the frontal cortex did not replicate these findings. Mosebach et al. [52] utilized OLIG1 as a distinguishing marker for OPCs and mature oligodendrocytes, leveraging its nuclear localization in OPCs and its cytoplasmic presence in mature oligodendrocytes. Using this method, they found no significant differences in OPC density between SCZ patients and controls in the pregenual anterior cingulate (BA32), dorsolateral prefrontal cortex (BA9), or the adjacent white matter [52]. Similarly, an independent study by Mauney et al., which employed CSPG4/NG2 as an OPC marker, reported no significant differences in OPC density in BA9 between SCZ patients and matched controls. These findings suggest that OPC density reductions are not a consistent feature of the frontal cortex in SCZ [52,53,54].

Interestingly, despite an apparent lack of OPC loss in the frontal cortex, one study found that OPCs in this region exhibited increased morphological complexity in SCZ patients [54]. The same research group, using preclinical models, proposed that this OPC hypertrophy may be linked to elevated expression of the truncated D3 and D7 isoforms of Disrupted in Schizophrenia 1 (DISC1), a well-known risk gene for SCZ [54,55]. However, whether this hypertrophic response ultimately leads to OPC death and contributes to the reduced OPC density observed in parietal regions remains an open question. Alternatively, it may represent a distinct, region-specific pathological adaptation that does not necessarily result in OPC loss.

Overall, the available histological studies highlight significant variability in OPC alterations across brain regions in SCZ. These inconsistencies raise the possibility that OPC pathology may be relevant only to a specific subset of SCZ patients, potentially those with a distinct symptom profile. Moreover, when OPC abnormalities are observed, they appear to predominantly affect their classical role in myelination, further supporting the hypothesis that white matter dysfunction is a key contributor to SCZ pathophysiology.

## 7. Bipolar Disorder (BD)

BD, also known as manic depression, is a chronic and severe mood disorder characterized by alternating episodes of depression, mania, and hypomania. The disorder is classified into different subtypes based on its longitudinal course and the presence of specific mood episodes. BD type I is diagnosed when at least one manic episode occurs, whereas BD type II is defined by the presence of at least one hypomanic and one major depressive episode. Cyclothymic disorder is diagnosed when individuals experience fluctuating hypomanic and depressive symptoms that do not meet the full diagnostic criteria for BD type I or II [33,56]. The estimated global lifetime prevalence of BD spectrum disorders is approximately 2.4%, with BD type I affecting men and women equally, while BD type II is more commonly diagnosed in women [56].

Although the precise etiology of BD remains unknown, both genetic predispositions and environmental factors are thought to contribute to its onset. Similarly to SCZ, BD is associated with a wide range of neuropathological alterations, including abnormalities in oligodendrocyte lineage cells and white matter integrity [57,58]. However, research specifically focusing on OPCs in BD is relatively scarce, and many findings parallel those observed in SCZ, suggesting that OPC dysfunction may contribute to shared psychiatric symptoms across these disorders.

One study utilizing bulk RNA-seq datasets found that BD patients exhibited reduced expression of DDR1 isoforms enriched in OPCs within the dorsolateral prefrontal cortex [48]. In addition, an investigation of the putamen in BD patients reported sex-specific differences in CSPG4/NG2 transcript levels, which were elevated in both male and female BD individuals [33]. However, a separate study revealed that OPC density was selectively reduced in the putamen of male BD patients, with no significant changes detected in female patients [51].

Regarding cortical regions, including BA32 and BA9, as well as the adjacent white matter, no significant differences in OPC density were found between BD patients and healthy controls [52]. Similarly to SCZ, the majority of OPC-related findings in BD have been investigated within the context of the classical myelinogenic function of these cells. This suggests that while OPC dysfunction may be implicated in BD, it is primarily linked to disruptions in myelination rather than other non-canonical OPC functions.

## 8. Major Depressive Disorder (MDD)

MDD is a prevalent and debilitating mood disorder affecting an estimated 15–18% of the global population over a lifetime. Epidemiological data indicate that MDD is nearly twice as common in women as in men [59,60]. The disorder can manifest at any stage of life, from adolescence to late adulthood, and is characterized by a range of symptoms, including persistent low mood, anhedonia, excessive feelings of guilt or worthlessness, suicidal ideation, and disturbances in appetite and sleep [60]. While the precise neurobiological mechanisms underlying MDD remain unclear, various biological systems have been implicated in its onset and progression. Additionally, exposure to adverse life events and psychosocial stressors has been identified as a major risk factor for the development of MDD [61,62].

Despite increasing recognition of the role of glial cells in psychiatric disorders, relatively few studies have specifically examined the involvement of OPCs in MDD. Investigations in this area have employed a variety of methodologies, including microarray analysis, RNA sequencing, and histological assessments, to explore potential OPC alterations associated with the disorder.

## 9. Gene Expression Alterations

Aston et al. [63] conducted a microarray-based gene expression analysis on post mortem temporal cortex samples from individuals with MDD and matched controls. Their findings revealed dysregulation across multiple biological pathways, including neurodevelopment, signal transduction, cell communication, and myelination. Of particular relevance to this narrative review, they identified significant downregulation of *SOX10* and *OLIG2*, two pan-lineage markers of oligodendrocytes, as well as reductions in several myelin-related transcripts [63]. These results suggest that OPC involvement in MDD may primarily relate to alterations in oligodendrocyte maturation and the canonical myelination pathway.

A single-nucleus transcriptomic study analyzing BA9 samples from male individuals with MDD who had died by suicide provided a novel perspective on OPCs in depression and, more broadly, in psychiatric disorders [64]. This study reported that nearly 50% of differentially expressed genes were concentrated in two specific cell populations: deep-layer excitatory neurons and immature OPCs [64]. Computational modeling further indicated a significant reduction in ligand–receptor interactions between these two cellular clusters, implying deficits in neuron–OPC communication and suggesting that non-canonical OPC functions may be implicated in MDD pathogenesis [64].

This dataset has since been utilized in multiple computational analyses, either independently [65,66,67] or in combination with other RNA sequencing datasets [66,68]. A summary of the key insights derived from these analyses is outlined below.

A sex-specific investigation incorporating the dataset from Nagy et al. [64], along with an additional dataset including female MDD samples, found that transcriptomic alterations were largely sex-dependent. Specifically, dysregulation in astrocytes, OPCs, and excitatory neurons was more pronounced in males with MDD, whereas transcriptomic changes in parvalbumin-expressing interneurons and microglia were predominantly observed in females [66].

Xie et al. [67] further refined the understanding of OPC involvement in MDD by defining four distinct developmental stages of the oligodendrocyte lineage based on gene expression patterns. Their analysis identified key genes associated with each developmental stage and highlighted markers that facilitate transitions between these stages. Notably, they demonstrated that gene clusters related to OPCs exhibited the strongest predictive value for MDD occurrence, emphasizing the importance of this cell type in MDD pathophysiology.

Additional analyses of the same dataset [64,67] led Kokkosis et al. [65] to identify a novel subset of oligodendrocytes specific to MDD, referred to as “immune-oligodendrocytes”. Complementary preclinical data suggest that these cells may play a regulatory role in microglial activity and influence myelin integrity [65].

Lastly, Zhou et al. [68] integrated bulk RNA sequencing, single-nucleus RNA sequencing, and DNA methylation data from MDD patients and matched controls [datasets: GSE102556, GSE88890, GSE144136, and GSE197622] [64,69,70,71]. Their findings revealed significant alterations in ion channel and glutamate receptor pathways, with these changes being particularly enriched in OPCs [68].

Although these findings collectively highlight OPC dysfunction in MDD, they are based primarily on a single patient cohort. To strengthen these observations, further replication studies using independent human cohorts, including both male and female samples, are necessary to validate the conclusions drawn from the Nagy et al. dataset [64].

## 10. Histological Alterations in Depression

Histological investigations have revealed region-specific OPC abnormalities in MDD. A study examining the putamen of male MDD patients reported a reduction in OPC density, while mature oligodendrocyte density remained unchanged [51]. However, an independent analysis found no significant differences in *CSPG4*/*NG2* mRNA levels in the putamen of MDD individuals compared to controls [29]. This discrepancy suggests that while OPC numbers may be reduced, the remaining OPCs could exhibit increased NG2 expression. Notably, preclinical studies have demonstrated that NG2 can be cleaved and released into the extracellular environment to support neuronal function and behavior [62]. Therefore, an upregulation of NG2 in OPCs within the putamen of MDD patients might represent a compensatory response involving non-canonical OPC functions that influence neuronal activity.

In cortical regions, an increase in OPC density (identified by nuclear OLIG1 expression) was detected in the white matter adjacent to BA32 and BA9 in MDD post mortem samples, whereas mature oligodendrocyte density (cytoplasmic OLIG1 expression) and MBP levels remained unchanged [52]. This suggests that OPC alterations in MDD may not directly impact myelin content through the traditional myelinogenic pathway. In contrast, a separate study employing OPC-specific PDGFRA markers reported a reduction in OPC density in the frontal cortex of MDD individuals compared to age-matched controls [72]. The authors proposed that this OPC loss could lead to decreased fibroblast growth factor 2 (FGF2) secretion, potentially disrupting astrocyte and neuronal function, thereby impairing overall brain homeostasis [72]. However, caution is warranted when interpreting these results due to methodological concerns. Notably, the PMI in the MDD cohort was nearly twice as long as in control samples (MDD: 23 ± 10 h; controls: 12 ± 6 h), raising the possibility that the observed OPC reduction could be influenced by tissue degradation rather than reflecting genuine pathological changes [72].

Given the well-documented impact of early-life adversity on brain development [73], three additional studies explored the relationship between childhood trauma and OPC alterations in MDD. These studies compared post mortem samples from MDD patients with and without a history of childhood abuse (MDD-CA) against control samples [74,75,76]. Collectively, their findings indicated that OPC density in BA9, BA11, BA12, BA24, and BA32 was unchanged across all groups [74,75,76], but they identified brain region-specific alterations in myelin content. Specifically, a reduction in MBP—suggestive of myelin loss—was observed in BA9 in both MDD and MDD-CA cases [75]. However, a reduction in OLIG2+ cell density and an increase in mature oligodendrocyte density were exclusive to MDD-CA individuals, indicating potential alterations in oligodendrocyte maturation dynamics linked specifically to early-life trauma [76]. Additionally, a decrease in SOX10+ cell density and modifications in myelin content were reported in BA24 and BA32 white matter [76].

Further investigations in MDD-CA patients revealed an increase in perineuronal net (PNN) density, along with a higher proportion of parvalbumin-expressing (PV) neurons ensheathed by PNNs in BA11-12 [74]. Moreover, OPC proximity to PV neurons and PNN-related gene expression in OPCs were elevated in MDD-CA compared to both the MDD and control groups, with both parameters correlating with PNN density [74]. These findings suggest that early-life trauma may induce OPC-related alterations that influence PNN formation and neuronal excitability.

Taken together, the available evidence supports a role for OPC dysfunction in the pathophysiology of MDD. Unlike SCZ and BD, where OPC pathology appears predominantly linked to the canonical myelination pathway, findings in MDD suggest the involvement of both myelination-related and non-canonical OPC functions. Whether these distinct OPC alterations are driven by early-life stress, differences in symptom profiles, or region-specific brain changes remains an open question that warrants further investigation.

**Figure 1:**
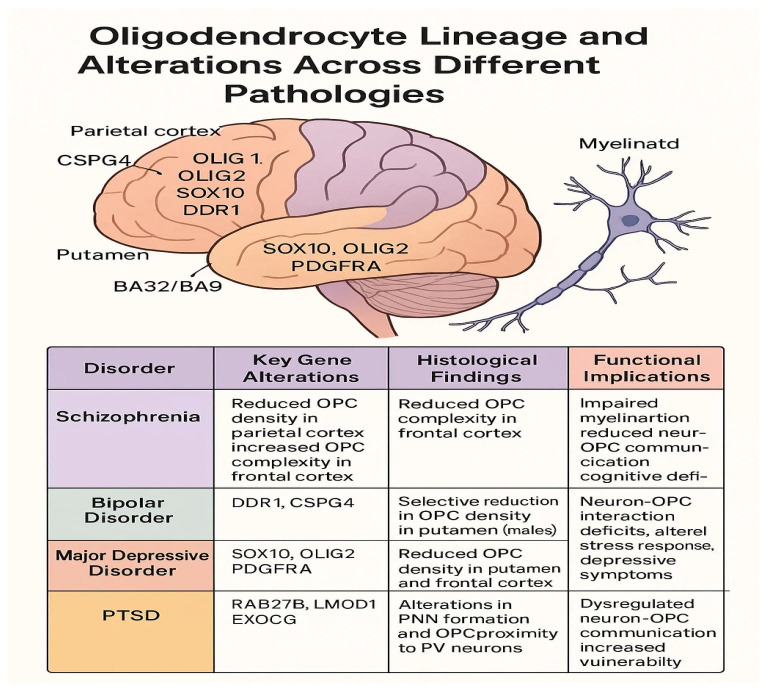
Oligodendrocytes lineage and alternation across different pathologies.

A visual summary of oligodendrocyte precursor cell alterations across major psychiatric disorders is provided in Figure 1, highlighting key gene dysregulations, regional histological findings, and their associated functional consequences.

## 11. Other Mental Disorders

In addition to SCZ, BD, and major MDD, three additional studies retrieved from our search focused on other psychiatric conditions [77] or adopted non-traditional diagnostic classifications to analyze multiple disorders [78,79].

One study specifically examined post-traumatic stress disorder (PTSD), a condition triggered by exposure to a severe traumatic event or prolonged stress [80]. PTSD symptoms can emerge immediately following trauma or remain latent for years before manifesting. They include heightened alertness, concentration, and sleep disturbances, emotional numbness, intrusive recollections or nightmares related to the trauma, and persistent feelings of guilt [33,80]. In this investigation, genome-wide association study (GWAS) data were integrated with findings from proteome-wide association studies (PWASs) and transcriptomic analyses, including microarray and single-cell RNA sequencing, to explore how genetic risk loci for PTSD influence mRNA as well as protein expression levels and increase vulnerability to the disorder [77]. The study identified seven genes strongly implicated in PTSD, all highly expressed in key brain regions involved in the disorder, such as the hippocampus, amygdala, cingulate cortex, and nucleus accumbens. Notably, three of these genes, Ras-Related Protein Rab-27B (*RAB27B*), Leiomodin 1 (*LMOD1*), and Exocyst Complex Component 6 (*EXOC6*), were enriched in OPCs and excitatory neurons. Among them, *EXOC6* emerged as the most robustly associated candidate. Given its established role in vesicular trafficking, this finding raises the possibility that PTSD pathology may involve disruptions in vesicle-mediated communication between excitatory neurons and OPCs [77].

Two other studies explored broader categories of psychiatric disorders using alternative classification approaches. One investigation focused on the effects of the *t(1:11)* chromosomal translocation, a genetic anomaly previously linked to various psychiatric conditions [78]. By combining hiPSC technology with transplantation techniques, the study demonstrated that *t(1:11)* translocation can lead to a reduction in proliferative OPCs while simultaneously increasing the density of O4+ oligodendrocytes. These changes were accompanied by alterations in oligodendrocyte- and myelin-related gene expression, as well as evidence of myelin loss [78].

The second study employed a novel classification framework to group psychiatric disorders into three broad categories: internalizing disorders (including depression, anxiety, and fear-related conditions), externalizing disorders (characterized by inattention, aggression, and disruptive behavior), and thought disorders (including delusions, hallucinations, and obsessive symptoms) [79]. A longitudinal analysis revealed that individuals with internalizing and externalizing disorders, but not those with thought disorders, exhibited increased cortical thickness. Interestingly, volume changes in the left caudal middle frontal gyrus in individuals with internalizing disorders were specifically associated with common genetic variations related to both OPCs and GABAergic neurons. This finding suggests that non-canonical OPC functions, particularly those involving neuron–OPC interactions, may be implicated in internalizing psychiatric conditions [79].

Collectively, these findings underscore the importance of further research into OPC pathology across a wider spectrum of mental illnesses. They highlight the potential involvement of OPCs beyond myelination, particularly in non-canonical pathways, such as synaptic regulation and neuron–glia communication, in psychiatric disorders.

## 12. Potential Role of Oligodendrocyte Precursor Cells in Psychiatry

This narrative review has compiled and analyzed the available evidence regarding the role of OPC dysfunction in the onset and progression of various psychiatric disorders, with a particular emphasis on clinical and post-mortem findings. To ensure a comprehensive and unbiased inclusion of relevant studies, broad search strategies were applied across two major databases. Despite the wide-ranging nature of these searches and the substantial number of retrieved studies, only a small fraction met the predefined inclusion criteria.

A bibliometric analysis revealed that research on OPC involvement in psychiatric disorders is still in its early stages, with the first study only published in 2003. Moreover, the majority of findings have been concentrated on two specific conditions: SCZ and MDD. One possible explanation for this narrow focus is the limited availability of post mortem brain samples from patients with other psychiatric conditions, despite the high prevalence of disorders such as anxiety. Nonetheless, existing data suggest that OPC dysfunction plays a role in psychiatric illnesses, even though the exact pathological mechanisms remain largely undefined.

In SCZ, most OPC-related alterations appear to be linked to disruptions in myelination, though recent findings also indicate potential impairments in non-canonical OPC functions, such as neuron–OPC communication and morphological abnormalities [48,54]. Findings in BD largely overlap with those observed in SCZ, implying that OPC dysfunction may contribute to shared symptomatology between these disorders. The picture in MDD is more complex; while several studies have reported OPC alterations associated with myelination deficits, others have highlighted changes affecting non-canonical OPC functions, including neuron–OPC interactions [64], brain homeostasis [72], and the formation or maintenance of perineuronal nets around parvalbumin interneurons [74].

Although the underlying mechanisms of OPC dysfunction in MDD may differ from those seen in SCZ, a common factor may be their timing. SCZ and BD typically manifest in early adulthood, while early-life stress has been identified as a major risk factor for MDD, suggesting that OPC dysfunction during critical developmental windows may play a key role in psychiatric vulnerability. Given the growing recognition of OPCs as fundamental regulators of brain circuit maturation [81,82], early disruptions in OPC function could lead to widespread impairments in cognitive and emotional processing later in life. This could represent a biological mechanism common to multiple psychiatric disorders, either as a primary driver of disease or as an exacerbating factor.

Within this developmental framework, it is also important to consider another cell type frequently implicated in psychiatric disorders: parvalbumin-positive (PV+) interneurons. This is particularly relevant given the shared developmental origins of OPCs and PV+ interneurons [83,84], which may be crucial for understanding disease mechanisms. PV+ interneurons have been implicated in SCZ pathology, where their density appears unchanged, but their mRNA expression is reduced [85,86], suggesting functional impairments in their plasticity and activity [87,88,89]. Notably, PV+ interneurons are among the most heavily myelinated interneurons in the cerebral cortex, and their myelination is critical for coordinating pyramidal neuron activity [90]. Disruptions in the reciprocal interactions between OPCs and PV+ interneurons could potentially trigger pathological cascades leading to different psychiatric disorders, though the specific mechanisms remain to be elucidated.

Future research efforts should address these knowledge gaps by integrating independent human cohort studies with robust preclinical models. Given the complexity of psychiatric disorders, as well as the overlap of symptoms and similarities in OPC-related alterations across multiple conditions, it would be advantageous to adopt transdiagnostic research frameworks, such as the Research Domain Criteria (RDoC) proposed by the National Institute of Mental Health [91]. By focusing on alterations in psychological and biological systems rather than rigid diagnostic categories, this approach may provide a more precise understanding of how OPC dysfunction contributes to specific symptom clusters.

Ultimately, elucidating the precise roles of OPCs in mental disorders could open new avenues for individualized therapeutic interventions. Moreover, harnessing the plasticity of OPCs may offer not only novel treatment strategies but also potential preventive approaches aimed at preserving mental health before the onset of psychiatric symptoms.

The main findings of this review are summarized in three tables at the end of the text: Table A1 presents gene expression alterations in psychiatric disorders, Table A2 outlines histological changes, and Table A3 provides a comprehensive analysis of OPC-related dysfunctions across different conditions.

## 13. Conclusions

The growing body of evidence suggests that OPCs play a significant role in the development and progression of major psychiatric disorders, particularly schizophrenia, bipolar disorder, and major depressive disorder. Beyond their well-established function in myelination, OPCs appear to influence neuronal communication, network plasticity, and brain homeostasis processes that are frequently disrupted in mental illness. While the current findings remain limited by methodological constraints and the narrow focus of existing studies, they underscore the importance of considering OPC dysfunction as a potential common denominator across distinct psychiatric conditions.

Future research should prioritize integrative approaches that combine clinical data, post mortem analyses, and experimental models, with special attention paid to developmental timing and transdiagnostic mechanisms. A better understanding of OPC contributions to brain circuit maturation and dysfunction may clarify disease mechanisms and open the door to innovative, targeted interventions, both therapeutic and preventive, to improve mental health outcomes.

## Appendix A. Summary of OPC Alterations in Psychiatric Disorders

Understanding the role of OPCs in psychiatric disorders requires an integrated approach that combines genetic, histological, and functional findings. The following tables summarize key findings from the literature review, highlighting gene expression changes, histopathological modifications, and their potential implications for brain function in different psychiatric conditions.

Table A1 presents the main gene expression alterations associated with OPC dysfunction in psychiatric disorders. These include the key genes involved, their expression patterns, and how these changes might impact oligodendrocyte lineage and myelination processes.

Table A2 focuses on histological changes observed in post mortem brain samples, specifying affected brain regions and the nature of OPC alterations. These findings provide insights into structural abnormalities linked to psychiatric disorders.

Table A3 offers a comprehensive overview, integrating gene expression changes, histological findings, and their functional implications. This table highlights the broader impact of OPC dysfunction on cognitive and emotional processing, emphasizing potential shared mechanisms across disorders.

**Table A1:** Gene expression alterations.

Authors	Disorder	Key Genes Altered	Expression Changes
Mauney et al. [53]	Schizophrenia	CSPG4, OLIG1, OLIG2, SOX10, DDR1	Reduced expression of oligodendrocyte-related genes, altered OPC differentiation
Barley et al. [29]	Bipolar Disorder	DDR1, CSPG4	Downregulation of myelin-related genes, sex-specific NG2 expression changes
Aston et al. [63]	Major Depressive Disorder	SOX10, OLIG2, PDGFRA	Reduction in SOX10/OLIG2 expression, altered neuron–OPC communication
Wingo et al. [77]	PTSD	RAB27B, LMOD1, EXOC6	Disruptions in vesicle-mediated communication between excitatory neurons and OPCs

This table summarizes the main gene expression alterations observed in the psychiatric disorders analyzed in the article. It lists the key genes involved, changes in their expression, and their potential impact on oligodendrocyte precursor cells (OPCs).

**Table A2:** Histological alterations.

Authors	Disorder	Histological Findings	Brain Regions Affected
Kolomeets et al. [50]	Schizophrenia	Reduced OPC density in the parietal cortex (BA39, BA40), increased OPC complexity in the frontal cortex	Parietal cortex (BA39, BA40), frontal cortex (BA9, BA32)
Kolomeets et al. [51]	Bipolar Disorder	Selective reduction in OPC density in the putamen of male BD patients	Putamen
Kolomeets et al. [51]	Major Depressive Disorder	Reduced OPC density in the putamen and frontal cortex, increased OPC density in BA32/BA9 white matter	Putamen, frontal cortex (BA32, BA9), and ventromedial prefrontal cortex
Wingo et al. [77]	PTSD	Alterations in perineuronal net (PNN) formation and OPC proximity to PV neurons	Hippocampus, amygdala, cingulate cortex, and nucleus accumbens

This table presents the main histological changes observed in post mortem brain tissues of individuals with psychiatric disorders. It includes key histological findings and the brain regions affected by OPC alterations.

**Table A3:** Comprehensive psychiatric OPC analysis.

Authors	Disorder	Key Gene Alterations	Histological Findings	Functional Implications
Kolomeets et al. [50]	Schizophrenia	CSPG4, OLIG1, OLIG2, SOX10, and DDR1	Reduced OPC density in the parietal cortex, increased OPC complexity in the frontal cortex	Impaired myelination, reduced neuron–OPC communication, and cognitive deficits
Kolomeets et al. [51]	Bipolar Disorder	DDR1, CSPG4	Selective reduction in OPC density in the putamen (males)	Disruptions in myelin maintenance, sex-dependent OPC dysfunction
Kolomeets et al. [51]	Major Depressive Disorder	SOX10, OLIG2, and PDGFRA	Reduced OPC density in the putamen and frontal cortex, increased OPC density in BA32/BA9 white matter	Neuron–OPC interaction deficits, altered stress response, and depressive symptoms
Wingo et al. [77]	PTSD	RAB27B, LMOD1, and EXOC6	Alterations in PNN formation and OPC proximity to PV neurons	Dysregulated neuron–OPC communication, increased vulnerability to stress-related disorders

This table provides an integrated view of oligodendrocyte precursor cell (OPC) alterations in psychiatric disorders, combining data on gene expression, histological modifications, and potential functional implications across the analyzed conditions.
